# Approaches to the Estimation of the Local Average Treatment Effect in a Regression Discontinuity Design

**DOI:** 10.1111/sjos.12224

**Published:** 2016-03-22

**Authors:** Aidan G. O'Keeffe, Gianluca Baio

**Affiliations:** ^1^ Department of Statistical ScienceUniversity College LondonLondonUK

**Keywords:** causal inference, local average treatment effect, regression discontinuity design, two‐stage least squares

## Abstract

Regression discontinuity designs (RD designs) are used as a method for causal inference from observational data, where the decision to apply an intervention is made according to a ‘decision rule’ that is linked to some continuous variable. Such designs are being increasingly developed in medicine. The local average treatment effect (LATE) has been established as an estimator of the intervention effect in an RD design, particularly where a design's ‘decision rule’ is not adhered to strictly. Estimating the variance of the LATE is not necessarily straightforward. We consider three approaches to the estimation of the LATE: two‐stage least squares, likelihood‐based and a Bayesian approach. We compare these under a variety of simulated RD designs and a real example concerning the prescription of statins based on cardiovascular disease risk score.

## 1 Introduction

Regression discontinuity designs (RD designs) have been developed as a method for causal inference in a variety of observational data settings (Berk & Leeuw, 1999; van der Klaauw, 2002, 2008; Lee, 2008). Such designs are quasi‐experimental and rely on the exploitation of situations where an intervention is assigned to subjects according to a pre‐specified rule (known as an intervention threshold) linked to a continuous variable (known as an assignment variable). A key idea behind an RD design is that subjects with similar assignment variable values might be considered as ‘exchangeable’. Under this assumption, we consider subjects whose assignment variables lie ‘just above’ the threshold to be similar to subjects whose assignment variable values lie ‘just below’ the threshold. A suitable comparison of outcomes between these two groups of subjects may be appropriate for an assessment of the causal effect of the intervention on some outcome variable of interest.

For example, consider a medical context in which an oral drug is prescribed with the aim of reducing a patient's blood pressure. Furthermore, the drug is prescribed only to those patients whose systolic blood pressure exceeds 140 mmHg. Here, the *intervention* is the oral drug for which a prescription is made according to a pre‐specified *intervention threshold* that is whether or not a patient's systolic blood pressure exceeds 140 mmHg. The systolic blood pressure is compared directly with the threshold and, as such, is the *assignment variable*. The outcome variable is the blood pressure measurement at some later point in time, after the decision of whether or not to prescribe the oral drug has been taken. The causal effect that we would like to measure is the effect of the oral drug on systolic blood pressure.

In many scenarios, an intervention threshold may not be adhered to strictly, resulting in some subjects receiving (or not receiving) the intervention contrary to what would be indicated by their assignment variable. This is known as a ‘fuzzy RD design’, and the estimation of the causal effect of the intervention must account for this ‘fuzziness’ present in the observed data. Typically, a local average treatment effect (LATE) estimator (Imbens & Angrist, 1994; Hahn et al., 2001) is used to provide an estimate of this effect.

Suppose that *Y* denotes the continuous outcome of interest and *Z*∈{0,1}, *T*∈{0,1} denote binary indicators of threshold attainment and intervention respectively, such that

$$Z=\left\{\begin{array}{cc} 1 & \text{if a subject attains the threshold;}\\ 0 & \text{otherwise.} \end{array}\right.$$

$$T=\left\{\begin{array}{cc} 1 & \text{if a subject receives the intervention;}\\ 0 & \text{otherwise.} \end{array}\right.$$

Then, the LATE at the threshold is defined as

(1) $$\text{LATE}=\frac{E(Y|Z=1)-E(Y|Z=0)}{P(T=1|Z=1)-P(T=1|Z=0)}.$$

It can be shown that the LATE gives an unbiased estimate of the intervention effect (see Hahn et al., 2001, and Section A of the Supporting Information for this paper), under certain assumptions, within a fuzzy RD design. If the intervention threshold rule is adhered to strictly, then the RD design is termed ‘sharp’, and the causal effect of the intervention on *Y* is recovered from the average treatment effect at the threshold (ATE) with

ATE=E(Y|Z=1)−E(Y|Z=0).

In an RD design, the threshold indicator, *Z*, can be seen as a special case of a binary instrumental variable (Angrist et al., 1996; Didelez et al., 2010). As such, the threshold might be considered as an instrumental variable for the intervention and the LATE (1) used to identify the causal effect of the intervention at the threshold in a population of *compliers*, that is, those individuals who are able to receive the intervention when their assignment variable moves from a point below the intervention threshold to a point above the intervention threshold. Furthermore, the LATE (1) is only valid within a population whose assignment variable values lie within a region close enough to the threshold for individuals to be considered exchangeable.

In econometrics, both the ATE and the LATE are typically estimated using a two‐stage least squares regression approach (Imbens & Angrist, 1994; Angrist & Imbens, 1995; Imbens & Lemieux, 2008). Using two‐stage least squares for estimation of the LATE can be advantageous, in that an unbiased estimator for the LATE is recovered and estimation may be performed relatively easily using standard statistical software. However, other approaches for the estimation of the LATE in an RD design can be taken. Two such approaches are maximum likelihood‐based or Bayesian estimation methods. Each of these methods relies on the specification and fitting of appropriate models for the numerator and denominator of the LATE.

With a maximum likelihood‐based approach, it is well known that the estimation of the LATE variance is not necessarily straightforward (Imbens & Lemieux, 2008). As a result, the two‐stage least squares approach has often been preferred, especially because an approximation for the variance of the LATE can be computed relatively easily. With a Bayesian approach, estimation of the LATE variance can be less problematic (Koo, 1997; Geneletti et al., 2015).

In this work, we aim to outline and compare these three approaches with LATE estimation (two‐stage least squares, maximum likelihood and Bayesian) within a fuzzy RD design. We focus on the fuzzy RD design because fuzziness is almost always present in observational data, especially in medical studies, making the fuzzy RD design more widely used. In a sharp RD design, the use of the ATE at the threshold makes treatment effect estimation more straightforward. In particular, we examine and compare estimation of the variance of the LATE for these three approaches.

This paper is organized as follows: in Section 2, we describe the RD design. In Section 3, we outline the three approaches to estimation of the LATE. In Section 4, a simulation study is presented in which the estimation methods described in Section 3 are performed under a variety of RD designs. Section 5 contains an example using real data on the prescription of statins in UK primary care according to the risk of cardiovascular disease (CVD). A discussion of results and conclusions is provided in Section 6.

## 2 The regression discontinuity design

An RD design can be used as a method to estimate the causal effect of a particular intervention using an observational dataset. In a population of N∈N subjects, we assume that information exists concerning the allocation of subjects to the intervention according to the subject‐specific value of a continuous ‘assignment variable’. The assignment variable is compared with a pre‐specified intervention ‘threshold’ whereby a subject receives the intervention if his or her assignment variable is greater than or equal to the intervention threshold value and does not receive the intervention if his or her assignment variable is less than the intervention threshold value.

The RD design uses the intervention threshold, and the assumption is made that subjects whose assignment variable values lie ‘just above’ or ‘just below’ the intervention threshold belong to the same population. As such, one might assume that the populations ‘just above’ and ‘just below’ the threshold are balanced with respect to unobserved confounders, allowing the causal effect of the intervention on the outcome of interest to be estimated. The RD design was first developed during the 1960s (Thistlethwaite & Campbell, 1960) and has been used extensively in economics (van der Klaauw, 2002, 2008; Jacob & Lefgren, 2004; Cellini et al., 2010; Anderson & Magruder, 2012). More recently, some researchers have begun to consider the use of the RD design to assess intervention effects in medicine (Linden et al., 2006; Rutter, 2009; Bor et al., 2014; O'Keeffe et al., 2014; Smith et al., 2015; Moscoe et al., 2015).

**Figure 1:**
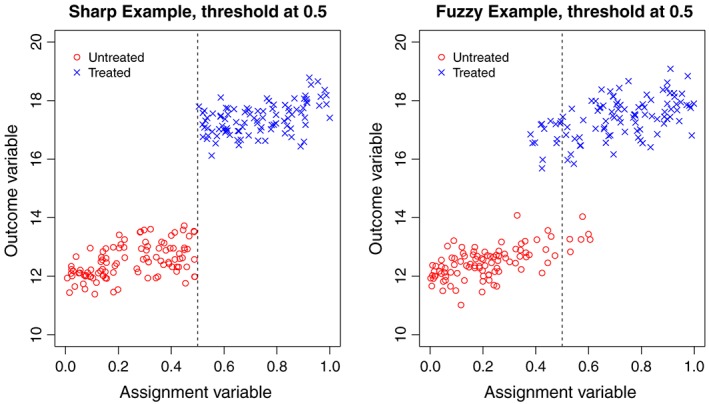
Example sharp and fuzzy RD design plots. The dashed vertical line represents the intervention threshold. ‘Untreated’ labels subjects who do not receive the intervention; ‘Treated’ labels subjects who receive the intervention.

There are two common forms of RD design. When the intervention rule (the threshold) is adhered to strictly, the design is known as sharp. In this scenario, all subjects whose assignment variable value lies at or above the threshold receive the intervention, and those whose assignment variable value lies below the threshold value do not receive the intervention. However, intervention thresholds are not always adhered to, in which case the RD design is known as fuzzy. In a fuzzy design, for some subjects, intervention assignment might be contrary to that indicated by the value of their assignment variable. Figure 1 shows example plots of an assignment variable (continuous from 0 to 1) against a hypothetical continuous outcome for sharp and fuzzy RD designs.

The left‐hand plot shows a sharp design, and the right‐hand plot shows a fuzzy design, and in each case, the intervention threshold occurs where the assignment variable = 0.5. An obvious discontinuity in the outcome variable exists at the intervention threshold. As noted previously, an assumption is made that subjects whose assignment variable values lie close to the intervention threshold are considered as coming from the same population. The notion of an assignment variable ‘lying close’ to the threshold is quantified by the choice of a pre‐specified bandwidth, *h*, such that only those subjects whose assignment variable values lie within a distance *h* of the intervention threshold are included in an RD design. We now define the variables of interest formally with the index *i*∈{1,…,*N*} denoting the subject.

### 2.1 Definitions and notation

Throughout, we assume that the assignment variable takes values in [0,1] although this assumption may be relaxed, without loss of generality, as long as the assignment variable is continuously distributed. We suppose that *x*_0_∈[0,1] represents the pre‐specified intervention threshold value and define the following variables:

$$\begin{array}{cc} X_{i} & \in \left[0,1\right]:\text{a continuous assignment variable.}\\ Z_{i} & =\left\{\begin{array}{cc} 0 & \text{if}X_{i}<x_{0}\\ 1 & \text{if}X_{i}\geq x_{0} \end{array}\right.\text{a threshold indicator;}\\ T_{i} & =\left\{\begin{array}{cc} 0 & \text{if the}\;i\text{th}\text{subject does not receive the intervention;}\\ 1 & \text{if the}\;i\text{th}\text{subject receives the intervention;} \end{array}\right.\\ Y_{i} & \in R:\text{a continuous outcome variable.} \end{array}$$

Furthermore, let *h*∈(0,1] denote the RD design bandwidth such that a subject's data are included in the design if *X*_*i*_∈(*x*_0_−*h*,*x*_0_+*h*).

As discussed in Section 1, we consider the LATE (1) as an estimator for the causal effect of the intervention at the threshold for fuzzy RD designs. It is the estimation of the LATE and its variance within a fuzzy RD design to which we give attention in this paper.

## 3 Estimation of the LATE in a fuzzy regression discontinuity design

We outline the three approaches to the estimation of the LATE and its variance: two‐stage least squares, maximum likelihood and Bayesian. The notation introduced in Section 2 is used together with the notation (*t*_*i*_,*x*_*i*_,*y*_*i*_,*z*_*i*_) as the observed counterparts of (*T*_*i*_,*X*_*i*_,*Y*_*i*_,*Z*_*i*_), and, furthermore, we make the assumption that the outcome variable, *Y*_*i*_, is normally distributed for each subject, a common assumption made when using an RD design with a continuous outcome variable. We note that the assumption of a normally distributed outcome variable is not a necessary condition for an RD design. Many applications of the RD design have used ordinary least squares for estimation purposes, particularly in econometrics, which can be more desirable than a maximum likelihood‐based approach if the assumption of a normally distributed outcome variable is not appropriate.

We consider a subpopulation of n<N∈N subjects to be included in our RD design and consider two subsets of subjects

$$\begin{array}{cc} A & =\left\{i|Z_{i}=1\cap X_{i}\in \left[x_{0},x_{0}+h\right)\right\}\\ ℬ & =\left\{i|Z_{i}=0\cap X_{i}\in \left(x_{0}-h,x_{0}\right)\right\} \end{array}$$

such that {1,…,n}=A∪ℬ. Essentially, A denotes the set of subjects whose assignment variables lie *above* the intervention threshold, and ℬ is the set of subjects whose assignment variables lie *below* the intervention threshold. In each case, we consider only those subjects whose assignment variables lie within a distance *h* of the threshold (i.e. those for whom *X*_*i*_∈(*x*_0_−*h*,*x*_0_+*h*)). We define $n_{a}=|A|$ and $n_{b}=|ℬ|$ to be the number of subjects above and below the threshold, respectively, whose data we consider for an RD design analysis. Given the threshold value *x*_0_, we define the ‘centred assignment variable’ as

$$X_{i}^{c}=X_{i}-x_{0}$$

with observed counterpart $x_{i}^{c}$. The use of a centred assignment variable shifts the threshold to zero and allows the LATE numerator to be defined using intercept parameters from linear models. Throughout, we denote the LATE at the threshold by the parameter *λ*.

### 3.1 Two‐stage least squares approach

As noted previously, the two‐stage least squares approach to the estimation of the LATE is often employed in RD designs (van der Klaauw, 2002; Imbens & Lemieux, 2008; Hoekstra, 2009). The approach is simple, relies on few assumptions concerning the variables defined in an RD design (i.e. the assignment variable, intervention indicator and outcome variable) and can be easily implemented using standard statistical software (Imbens & Lemieux, 2008).

In the two‐stage least squares approach, we fit two linear models. First, we regress *T*_*i*_ on *z*_*i*_ and $x_{i}^{c}$ using a model of the form

(2) $$T_{i}=\alpha_{0}+\alpha_{1}z_{i}+\alpha_{2}(1-z_{i})x_{i}^{c}+\alpha_{3}z_{i}x_{i}^{c}+\omega_{1i}$$

with *ω*_1*i*_(*i*∈{1,…,*n*}) denoting independent mean zero error terms. The model (2) is fitted using least squares, and the vector of fitted values $\hat{t}={\left({\hat{t}}_{i},\ldots ,{\hat{t}}_{n}\right)}^{⊤}$ is extracted. These fitted values are used as explanatory variables in a linear model for the outcome *Y*_*i*_, of the form

$$Y_{i}=\gamma_{0}+\gamma_{1}{\hat{t}}_{i}+\gamma_{2}(1-{\hat{t}}_{i})x_{i}^{c}+\gamma_{3}{\hat{t}}_{i}x_{i}^{c}+\omega_{2i}$$

with *ω*_2*i*_ independent mean zero error terms. To fit these models, we define the following design matrices:

$$Ž=\left(\begin{array}{cccc} 1 & z_{1} & (1-z_{1})x_{1} & z_{1}x_{1}\\ \vdots & \vdots & \vdots & \vdots \\ 1 & z_{n} & (1-z_{n})x_{n} & z_{n}x_{n} \end{array}\right)$$

and

(3) $$\overset{ˇ}{X}=\left(\begin{array}{cccc} 1 & {\hat{t}}_{1} & (1-{\hat{t}}_{1})x_{1}^{c} & {\hat{t}}_{1}x_{1}^{c}\\ \vdots & \vdots & \vdots & \vdots \\ 1 & {\hat{t}}_{n} & (1-{\hat{t}}_{n})x_{n}^{c} & {\hat{t}}_{n}x_{n}^{c} \end{array}\right).$$

We write the models in matrix form as

$$\begin{array}{cc} T & =Ž\alpha +\omega_{1}\\ Y & =\overset{ˇ}{X}\gamma +\omega_{2} \end{array}$$

with ***ω***_1_ and ***ω***_2_ assumed independent. The least squares estimates of ***α*** = (*α*_0_,*α*_1_,*α*_2_,*α*_3_)^⊤^ are

$$\hat{\alpha }={\left({Ž}^{⊤}Ž\right)}^{-1}{Ž}^{⊤}T$$

and so the corresponding fitted values ${\left({\hat{t}}_{1},\ldots ,{\hat{t}}_{n}\right)}^{⊤}$ are given by

$${\left({\hat{t}}_{1},\ldots ,{\hat{t}}_{n}\right)}^{⊤}=Ž{\left({Ž}^{⊤}Ž\right)}^{-1}{Ž}^{⊤}T.$$

In addition, the least squares estimates of $\hat{\gamma }={\left(\gamma_{0},\gamma_{1},\gamma_{2},\gamma_{3}\right)}^{⊤}$ are

(4) $$\hat{\gamma }={\left({\overset{ˇ}{X}}^{⊤}\overset{ˇ}{X}\right)}^{-1}{\overset{ˇ}{X}}^{⊤}Y.$$

The intervention effect estimate at the threshold is given by ${\hat{\gamma }}_{1}$, and the variance–covariance matrix of $\hat{\gamma }$ is

$$\begin{array}{cc} \mathrm{Var}(\hat{\gamma }) & =\mathrm{Var}(Y){\left({\overset{ˇ}{X}}^{⊤}\overset{ˇ}{X}\right)}^{-1}\\ =\sigma^{2}{\left({\overset{ˇ}{X}}^{⊤}\overset{ˇ}{X}\right)}^{-1}. \end{array}$$

So the LATE variance might be given by

(5) $$\mathrm{Var}({\hat{\gamma }}_{1})={\left[\sigma^{2}{\left({\overset{ˇ}{X}}^{⊤}\overset{ˇ}{X}\right)}^{-1}\right]}_{22}.$$

That is, the (2,2) element of the variance–covariance matrix for ***γ*** because ${\hat{\gamma }}_{1}$ denotes the LATE at the threshold in this model. Typically, *σ* might be estimated using the residual sum of squares from the fitted model

$${\hat{\sigma }}^{2}=\frac{1}{n-4}∥Y-\overset{ˇ}{X}\hat{\gamma }{∥}^{2},$$

with a denominator of *n* − 4 because ***γ*** contains four parameters. A standard estimate of the variance of the LATE would be given by

(6) $${\mathrm{Var}}^{\text{std}}(\hat{\lambda })={\left[{\hat{\sigma }}^{2}{\left({\overset{ˇ}{X}}^{⊤}\overset{ˇ}{X}\right)}^{-1}\right]}_{22}$$

where $\hat{\lambda }={\hat{\gamma }}_{1}$. However, it is known that this approach will lead to a loss in efficiency in the estimate of the LATE variance from two‐stage least squares (Baltagi, 2011). To reduce the loss in efficiency, it is suggested that the residual sum of squares from the fitted model ( $∥Y-\overset{ˇ}{X}\hat{\gamma }{∥}^{2}$) is adjusted by replacing the fitted ${\hat{t}}_{i}$ values in the design matrix (3) with the actual values *t*_*i*_. We define the adjusted design matrix:

(7) $$\tilde{X}=\left(\begin{array}{cccc} 1 & t_{1} & (1-t_{1})x_{1}^{c} & t_{1}x_{1}^{c}\\ \vdots & \vdots & \vdots & \vdots \\ 1 & t_{n} & (1-t_{n})x_{n}^{c} & t_{n}x_{n}^{c} \end{array}\right)$$

and the adjusted estimate of *σ* ^2^ is given by

(8) $${\tilde{\sigma }}^{2}=\frac{1}{n-4}∥Y-\tilde{X}\hat{\gamma }{∥}^{2}.$$

The adjusted variance estimate of the LATE, under two‐stage least squares estimation, is given by

(9) $${\mathrm{Var}}^{\text{adj}}(\hat{\lambda })={\left[{\tilde{\sigma }}^{2}{\left({\overset{ˇ}{X}}^{⊤}\overset{ˇ}{X}\right)}^{-1}\right]}_{22}.$$

With this method, the estimate for *σ* ^2^ has been artificially adjusted, and, consequently, comparison with the estimate of *σ* ^2^ used in ${\mathrm{Var}}^{\text{std}}(\hat{\lambda })$ (or with the estimates used in maximum likelihood and Bayesian approaches) is not straightforward.

Hence, we have a method for the unbiased estimation of the LATE, together with two methods for variance estimation ( ${\mathrm{Var}}^{\text{std}}(\hat{\lambda })$ and ${\mathrm{Var}}^{\text{adj}}(\hat{\lambda })$) using a two‐stage least squares approach. We now consider a maximum likelihood‐based approach to the estimation of the LATE.

### 3.2 Maximum likelihood‐based approach

Unlike the two‐stage least squares approach to estimation of the LATE, the maximum likelihood‐based approach relies directly on distributional assumptions of both the outcomes *Y*_1_,…,*Y*_*n*_ and binary treatment variables *T*_1_,…,*T*_*n*_. Under the assumption that each *Y*_*i*_ is, independently, normally distributed, we construct the following normal linear models for *Y*_1_,…,*Y*_*n*_:

(10) $$\begin{array}{cc} Y_{i} & =\beta_{0a}+\beta_{1a}x_{i}^{c}+\varepsilon_{i}\text{for}i\in A\\ Y_{i} & =\beta_{0b}+\beta_{1b}x_{i}^{c}+\varepsilon_{i}\text{for}i\in ℬ \end{array}$$

with $\varepsilon_{i}∼N(0,\sigma^{2})$ independently for *i*∈{1,…,*N*}. We note that, at the threshold (i.e. where $x_{i}^{c}=0$),

$$\begin{array}{cc} E\left(Y_{i}|Z_{i}=1\right)-E\left(Y_{i}|Z_{i}=0\right) & =\beta_{0a}-\beta_{0b}\\ =\beta . \end{array}$$

Next, we consider a model for *T*_*i*_(*i*∈{1,…,*n*}). Conditional on *Z*_*i*_ and $X_{i}^{c}$, the probability of treatment receipt is modelled as

$$\begin{array}{cc} P\left(T_{i}=1|Z_{i}=1,X_{i}^{c}\right) & =\pi_{0a}+\pi_{1a}x_{i}^{c}\text{if}i\in A;\\ P\left(T_{i}=1|Z_{i}=0,X_{i}^{c}\right) & =\pi_{0b}+\pi_{1b}x_{i}^{c}\text{if}i\in ℬ. \end{array}$$

Then, at the threshold, we have

$$E\left(T|Z=1\right)-E\left(T|Z=0\right)=\pi_{0a}-\pi_{0b}=\pi$$

and the LATE at the threshold is written

$$\text{LATE}=\lambda =\frac{\beta }{\pi }.$$

We consider separate maximum likelihood estimation of *β* and *π*. The maximum likelihood estimators for (*β*_0*a*_,*β*_0*b*_)^⊤^ and (*π*_0*a*_,*π*_0*b*_)^⊤^, denoted ${\left({\hat{\beta }}_{0a},{\hat{\beta }}_{0b}\right)}^{⊤}$ and ${\left({\hat{\pi }}_{0a},{\hat{\pi }}_{0b}\right)}^{⊤}$, respectively, can be easily obtained (with their explicit form given in Section B of the Supporting Information for this paper). Using the invariance property for maximum likelihood estimators, the maximum likelihood estimators for the LATE numerator and denominator (at the threshold) are written

$$\begin{array}{cc} \hat{\beta } & ={\hat{\beta }}_{0a}-{\hat{\beta }}_{0b};\\ \hat{\pi } & ={\hat{\pi }}_{0a}-{\hat{\pi }}_{0b} \end{array}$$

and the maximum likelihood estimator for the LATE is

$$\hat{\lambda }=\frac{\hat{\beta }}{\hat{\pi }}.$$

We see that the maximum likelihood estimator for the LATE is formed as a ratio of two other estimators. As such, computation of the variance of the LATE maximum likelihood estimator is not straightforward. Using a Taylor series approximation, with a full derivation provided in Section B of the Supporting Information, we form the following estimate for the variance of the LATE maximum likelihood estimator:

(11) $$\begin{array}{cc} \mathrm{Var}(\hat{\lambda }) & =\frac{{\hat{\sigma }}^{2}}{{\hat{\pi }}^{2}}\left(\underset{i\in A}{\sum }a_{i}^{2}+\underset{i\in ℬ}{\sum }b_{i}^{2}\right)+\frac{{\hat{\beta }}^{2}}{{\hat{\pi }}^{4}}\left({\hat{\phi }}_{a}^{2}\underset{i\in A}{\sum }a_{i}^{2}+{\hat{\phi }}_{b}^{2}\underset{i\in ℬ}{\sum }b_{i}^{2}\right)\\ \;-\frac{2\hat{\beta }}{{\hat{\pi }}^{3}}\left({\hat{\rho }}_{a}\underset{i\in A}{\sum }a_{i}^{2}+{\hat{\rho }}_{b}\underset{i\in ℬ}{\sum }b_{i}^{2}\right) \end{array}$$

with

$$\begin{array}{cc} a_{i} & =\frac{1}{n_{a}}+\frac{1}{\underset{i\in A}{\sum }{\left(x_{\mathrm{ia}}^{c}-{\bar{x}}_{a}^{c}\right)}^{2}}\left({\left({\bar{x}}_{a}^{c}\right)}^{2}-{\bar{x}}_{a}^{c}x_{i}^{c}\right);\\ b_{i} & =\frac{1}{n_{b}}+\frac{1}{\underset{i\in ℬ}{\sum }{\left(x_{\mathrm{ib}}^{c}-{\bar{x}}_{b}^{c}\right)}^{2}}\left({\left({\bar{x}}_{b}^{c}\right)}^{2}-{\bar{x}}_{b}^{c}x_{i}^{c}\right);\\ s_{a}^{2} & =\frac{1}{n_{a}-2}\underset{i\in A}{\sum }{\left(y_{i}-{ŷ}_{i}\right)}^{2};\\ s_{b}^{2} & =\frac{1}{n_{b}-2}\underset{i\in ℬ}{\sum }{\left(y_{i}-{ŷ}_{i}\right)}^{2};\\ {\hat{\sigma }}^{2} & =\frac{(n_{a}-2)s_{a}^{2}+\left(n_{b}-2\right)s_{b}^{2}}{n_{a}+n_{b}-4};\\ {\hat{\phi }}_{a}^{2} & =\frac{1}{n_{a}-2}\underset{i\in A}{\sum }{\left(t_{i}-{\hat{t}}_{i}\right)}^{2};\\ {\hat{\phi }}_{b}^{2} & =\frac{1}{n_{b}-2}\underset{i\in ℬ}{\sum }{\left(t_{i}-{\hat{t}}_{i}\right)}^{2};\\ {\hat{\rho }}_{a} & =\frac{1}{n_{a}-1}\underset{i\in A}{\sum }\left(y_{i}-{ŷ}_{i}\right)\left(t_{i}-{\hat{t}}_{i}\right);\\ {\hat{\rho }}_{b} & =\frac{1}{n_{b}-1}\underset{i\in ℬ}{\sum }\left(y_{i}-{ŷ}_{i}\right)\left(t_{i}-{\hat{t}}_{i}\right). \end{array}$$

We notethat ${\bar{x}}_{j}^{c}$ denotes the sample mean of the assignment‐centred variable values above the threshold (*j* = *a*) or below the threshold (*j* = *b*). In addition, ${ŷ}_{i}$ and ${\hat{t}}_{i}$ denote the fitted values of the outcome variable and intervention indicator, for the *i*th subject. We now consider the Bayesian approach to estimation of the LATE at the threshold.

### 3.3 Bayesian approach

In a Bayesian framework, we consider the linear models (10) specified for the outcomes *Y*_*i*_,…,*Y*_*n*_ and place prior distributions on the model parameters (*β*_0*a*_,*β*_1*a*_,*β*_0*b*_,*β*_1*b*_,*σ* ^2^)^⊤^. Typically, we choose the prior distribution of each linear parameter (*β*_0*a*_,*β*_1*a*_,*β*_0*b*_,*β*_1*b*_) to be normal, whilst a probability distribution with support on a subset of (0,*∞*), such as the inverse gamma distribution or appropriate continuous uniform distribution, can be defined as the prior distribution for *σ* ^2^. For the intervention variables, we assume that *T*_*i*_∼Bin(1,*p*_*ai*_) for i∈A and *T*_*i*_∼Bin(1,*p*_*bi*_) for i∈ℬ with

$$\begin{array}{cc} \log\left(\frac{p_{\mathrm{ai}}}{1-p_{\mathrm{ai}}}\right) & =\gamma_{0}+\gamma_{1}x_{i}^{c}\\ \log\left(\frac{p_{\mathrm{bi}}}{1-p_{\mathrm{bi}}}\right) & =\gamma_{2}+\gamma_{3}x_{i}^{c} \end{array}$$

and normal priors placed on *γ*_0_,*γ*_1_,*γ*_2_ and *γ*_3_.

The Bayesian models are fitted using Markov chain Monte Carlo methods, easily applied using standard statistical software. The LATE at the threshold is estimated through computation of the posterior distribution of

$$\lambda =\frac{\beta_{0a}-\beta_{0b}}{\pi_{0a}-\pi_{0b}},$$

where

$$\begin{array}{cc} \pi_{0a} & =\frac{\exp(\gamma_{0})}{1+\exp(\gamma_{0})};\\ \pi_{0b} & =\frac{\exp(\gamma_{2})}{1+\exp(\gamma_{2})}. \end{array}$$

Because the probability distribution of *λ* is estimated or derived, information on uncertainty surrounding *λ*, including an estimate of Var(*λ*), can be easily obtained.

With a few exceptions (for example, Koo, 1997; Lee & Card, 2008; Geneletti et al., 2015), Bayesian methods have not been used extensively in RD designs. In many cases, a Bayesian approach is appealing. Firstly, prior information regarding the likely values of parameters used in the construction of the LATE can be incorporated into the modelling. For example, previous studies or research may have given insight on appropriate values of *β*_0*a*_ and *β*_0*b*_, together with possible information on the level of fuzziness that might be expected in the design, which could be incorporated into the choice of prior distributions (Geneletti et al., 2015). This may be particularly relevant where information about the efficacy of a treatment is known from a randomized trial conducted on a small population. Such information could be used in a prior distribution for an RD design analysis where the aim might be to assess the efficacy of the same treatment in a larger, more general, population from observational data. In addition, a Bayesian approach could allow for more flexible modelling assumptions than either the two‐stage least squares or maximum likelihood‐based methods.

In the next section, we introduce a simulation study in which the three methods of LATE estimation are compared.

## 4 Simulation study

We aim for the simulated data to be representative of data that might be observed in a fuzzy RD design. Suppose that we wish to simulate M∈N datasets, each of which contains data from N∈N subjects. The simulation algorithm is defined as follows:

1. Simulate *N* assignment variables **X** = (*X*_1_,…,*X*_*N*_)^⊤^ where each *X*_*i*_ is drawn at random from a standard uniform distribution ( U(0,1)). The threshold is set at 0.5 with the chosen bandwidth denoted *h*∈(0,1]. We denote vector of centred assignment variables:
  $$X^{c}={\left(X_{1}-0.5,\ldots ,X_{N}-0.5\right)}^{⊤}.$$
2. Define the threshold indicator:
  $$Z_{i}=\left\{\begin{array}{cc} 0 & \text{if}X_{i}^{c}<0\\ 1 & \text{if}X_{i}^{c}\geq 0 \end{array}\right.$$
  and the sets
  $$\begin{array}{cc} A & =\left\{i|Z_{i}=1\cap X_{i}^{c}\in [0,h)\right\};\\ ℬ & =\left\{i|Z_{i}=0\cap X_{i}^{c}\in \left(-h,0\right)\right\}. \end{array}$$
3. Draw the intervention indicators *T*_*i*_(*i*∈{1,…,*N*}) randomly as follows:
  $$T_{i}∼\left\{\begin{array}{cc} \text{Bin}(1,1-p) & \text{if}i\in A\\ \text{Bin}(1,p) & \text{if}i\in ℬ \end{array}\right.$$
  for a chosen ‘probability of non‐adherence’ *p*. Here, the term ‘probability of non‐adherence’ refers to the probability that the intervention rule is not followed for a given subject.
4. Draw the outcome *Y*_*i*_(*i*∈{1,…,*N*}) randomly as follows, conditional on $X_{i}^{c}=x_{i}^{c}$:
  $$Y_{i}∼\left\{\begin{array}{cc} N(5+0.4x_{i}^{c},1) & \text{if}T_{i}=0\\ N(3+0.3x_{i}^{c},1) & \text{if}T_{i}=1. \end{array}\right.$$
5. Repeat the aforementioned steps *M* times, thereby creating *M* simulated datasets.

**Figure 2:**
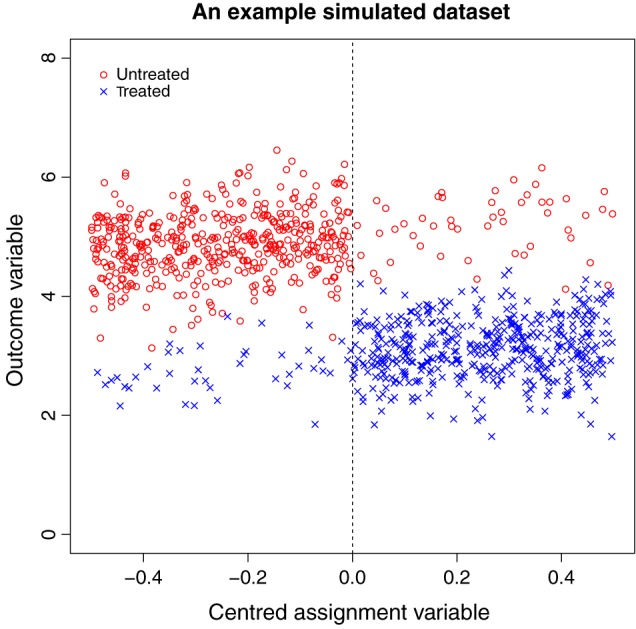
Example plot of a simulated dataset where *N* = 1000. The discontinuity in outcome variable at the threshold can be clearly observed. Red icons (marked ‘Untreated’) indicate subjects who do not receive the intervention, and blue icons (marked ‘Treated’) indicate subjects who receive the intervention. The dashed vertical line represents the intervention threshold.

Within each of these *M* datasets, the intervention effect at the threshold has size −2. Figure 2 shows an example plot of *x* ^*c*^ against *y* for a simulated dataset where *N* = 1000.

Using each dataset, we estimated both the LATE and its variance, using the methods described in Section 3. We term these variance estimation methods 2SLS (two‐stage least squares), MLE (maximum likelihood estimation) and BAYES (Bayesian estimation) with the corresponding LATE estimates denoted ${\hat{\lambda }}_{\text{2SLS}}$, ${\hat{\lambda }}_{\text{MLE}}$ and ${\hat{\lambda }}_{\text{BAYES}}$, respectively. For the two‐stage least squares approach, we define ${\hat{\nu }}_{\text{2SLS}}^{\text{std}}$ to be the ‘standard’ method of variance estimation using two‐stage least squares (6) and ${\hat{\nu }}_{\text{2SLS}}^{\text{adj}}$ to be the ‘adjusted’ method of variance estimation using a two‐stage least squares approach (9). We define ${\hat{\nu }}_{\text{MLE}}$ to be the LATE variance estimate using the MLE method and ${\hat{\nu }}_{\text{BAYES}}$ to be that using the Bayesian approach. For the Bayesian analysis, vague priors were assumed for *σ* ^2^, *β*_0*a*_, *β*_0*b*_, *β*_1*a*_, *β*_1*b*_, *γ*_0_, *γ*_1_, *γ*_2_ and *γ*_3_ (details given in Section C of the Supporting Information). We chose vague priors so that we would be able to ascertain the performance of the Bayesian method where prior influence was not too strong. However, we recognize that, in many scenarios, less vague prior distributions could be specified for parameters of interest (for example, Geneletti et al., 2015).

As a comparison, we calculated the sample variance of the *M* point estimates of *λ* in the frequentist and two‐stage least squares scenarios and denote these estimates as $V({\hat{\lambda }}_{\text{MLE}})$ and $V({\hat{\lambda }}_{\text{2SLS}})$, respectively. We chose varying dataset sizes of *N* = 250,500,1000and5000, for each of the two non‐adherence probabilities (*p* = 0.1or0.2), and repeated the simulation process *M* = 200 times for each dataset size and adherence probability. The choice of treatment effect size of −2 at the threshold was based loosely on a possible treatment effect for statins on low‐density lipoprotein (LDL) cholesterol level and is similar to that chosen in Geneletti *et al.* (2015). Results where the non‐adherence probability is 0.1 are given in Table 1, and those where the non‐adherence probability is 0.2 are given in Table 2.

**Table 1:** Two‐stage least squares estimates ( ${\hat{\lambda }}_{\text{2SLS}}$), maximum likelihood estimates ( ${\hat{\lambda }}_{\text{MLE}}$) and Bayesian estimates ( ${\hat{\lambda }}_{\text{BAYES}}$) of the LATE, together with corresponding variance estimates from the simulated datasets with a variety of chosen RD design bandwidths and dataset sizes

*h*	${\hat{\lambda }}_{\text{2SLS}}$	${\hat{\lambda }}_{\text{MLE}}$	${\hat{\lambda }}_{\text{BAYES}}$	${\hat{\nu }}_{\text{2SLS}}^{\text{std}}$	${\hat{\nu }}_{\text{2SLS}}^{\text{adj}}$	${\hat{\nu }}_{\text{MLE}}$	${\hat{\nu }}_{\text{BAYES}}$	V( ${\hat{\lambda }}_{\text{MLE}}$)	V( ${\hat{\lambda }}_{\text{2SLS}}$)
*Sample size = 5000, non‐adherence probability = 0.1*									
0.05	−2.00	−2.00	−2.01	0.0316	0.0130	0.0131	0.0191	0.0155	0.0153
0.10	−2.00	−2.00	−2.00	0.0156	0.0064	0.0064	0.0167	0.0058	0.0058
0.15	−2.00	−2.00	−2.01	0.0103	0.0042	0.0042	0.0137	0.0041	0.0041
0.20	−2.00	−2.00	−2.01	0.0077	0.0032	0.0032	0.0113	0.0031	0.0031
0.25	−2.00	−2.00	−2.01	0.0061	0.0025	0.0025	0.0094	0.0023	0.0023
*Sample size = 1000, non‐adherence probability = 0.1*									
0.05	−2.00	−2.00	−2.03	0.1727	0.0729	0.0775	0.0741	0.0683	0.0662
0.10	−2.00	−2.00	−2.02	0.0807	0.0334	0.0343	0.0552	0.0294	0.0288
0.15	−2.00	−2.00	−2.01	0.0532	0.0219	0.0225	0.0514	0.0261	0.0261
0.20	−2.00	−2.00	−2.01	0.0395	0.0162	0.0165	0.0465	0.0194	0.0193
0.25	−2.00	−2.00	−2.02	0.0314	0.0128	0.0130	0.0421	0.0146	0.0145
*Sample size = 500, non‐adherence probability = 0.1*									
0.05	−1.99	−1.99	−2.07	0.4251	0.1948	0.2274	0.1541	0.1712	0.1498
0.10	−2.00	−1.99	−2.04	0.1751	0.0755	0.0802	0.0965	0.0715	0.0692
0.15	−2.00	−2.00	−2.04	0.1104	0.0462	0.0479	0.0888	0.0471	0.0461
0.20	−2.00	−2.00	−2.02	0.0811	0.0340	0.0349	0.0826	0.0370	0.0363
0.25	−2.00	−2.00	−2.03	0.0640	0.0268	0.0274	0.0764	0.0299	0.0296
*Sample size = 250, non‐adherence probability = 0.1*									
0.05	−1.98	−1.96	−2.13	2.4527	25.2280	50.0041	6.3297	0.9389	0.6909
0.10	−1.96	−1.96	−2.08	0.4048	0.2020	0.2171	0.1880	0.1715	0.1684
0.15	−1.99	−1.99	−2.06	0.2360	0.1007	0.1104	0.1528	0.0992	0.0977
0.20	−2.01	−2.00	−2.06	0.1746	0.0734	0.0776	0.1443	0.0769	0.0757
0.25	−2.01	−2.01	−2.07	0.1351	0.0560	0.0583	0.1392	0.0611	0.0584

The non‐adherence probability is set at 0.1. $\text{V}({\hat{\lambda }}_{\text{MLE}})$ denotes the sample variance of the maximum likelihood estimates, and $\text{V}({\hat{\lambda }}_{\text{2SLS}})$ denotes the sample variance of the two‐stage least square estimates.

LATE, local average treatment effect; RD, regression discontinuity.

**Table 2:** Two‐stage least squares estimates ( ${\hat{\lambda }}_{\text{2SLS}}$), maximum likelihood estimates ( ${\hat{\lambda }}_{\text{MLE}}$) and Bayesian estimates ( ${\hat{\lambda }}_{\text{BAYES}}$) of the LATE, together with corresponding variance estimates from the simulated datasets with a variety of chosen RD design bandwidths and dataset sizes

*h*	${\hat{\lambda }}_{\text{2SLS}}$	${\hat{\lambda }}_{\text{MLE}}$	${\hat{\lambda }}_{\text{BAYES}}$	${\hat{\nu }}_{\text{2SLS}}^{\text{std}}$	${\hat{\nu }}_{\text{2SLS}}^{\text{adj}}$	${\hat{\nu }}_{\text{MLE}}$	${\hat{\nu }}_{\text{BAYES}}$	V( ${\hat{\lambda }}_{\text{MLE}}$)	V( ${\hat{\lambda }}_{\text{2SLS}}$)
*Sample size = 5000, non‐adherence probability = 0.2*									
0.05	−2.00	−2.00	−1.99	0.0851	0.0244	0.0248	0.0499	0.0240	0.0239
0.10	−2.01	−2.01	−2.00	0.0415	0.0117	0.0118	0.0455	0.0122	0.0123
0.15	−2.01	−2.01	−2.00	0.0272	0.0076	0.0077	0.0392	0.0084	0.0083
0.20	−2.01	−2.01	−2.01	0.0205	0.0058	0.0058	0.0325	0.0062	0.0062
0.25	−2.01	−2.01	−2.00	0.0163	0.0046	0.0046	0.0270	0.0050	0.0050
*Sample size = 1000, non‐adherence probability = 0.2*									
0.05	−1.97	−1.96	−1.95	0.7070	0.3314	0.4726	0.2008	0.2318	0.1860
0.10	−1.98	−1.97	−1.96	0.2366	0.0732	0.0769	0.1419	0.0759	0.0721
0.15	−1.99	−1.99	−1.97	0.1479	0.0437	0.0452	0.1361	0.0464	0.0457
0.20	−1.99	−1.99	−1.97	0.1078	0.0315	0.0321	0.1282	0.0347	0.0339
0.25	−2.00	−2.00	−1.98	0.0846	0.0245	0.0249	0.1171	0.0268	0.0268
*Sample size = 500, non‐adherence probability = 0.2*									
0.05	−2.12	−1.99	−1.92	2.8039	19.6660	398.6478	0.4603	0.8190	0.7911
0.10	−1.98	−1.98	−1.97	0.4996	0.1693	0.1997	0.2328	0.1608	0.1455
0.15	−2.01	−2.00	−1.98	0.2935	0.0888	0.0959	0.2175	0.0826	0.0767
0.20	−2.02	−2.01	−1.98	0.2190	0.0655	0.0678	0.2129	0.0613	0.0598
0.25	−2.02	−2.02	−1.99	0.1707	0.0505	0.0519	0.2031	0.0498	0.0481
*Sample size = 250, non‐adherence probability = 0.2*									
0.05	−2.02	−2.05	−1.93	7.0890	63.3852	412.9690	104.0435	5.3853	2.6314
0.10	−2.19	−2.17	−1.94	2.9853	99.5390	545.0271	0.5564	2.2327	3.2587
0.15	−2.01	−2.01	−1.96	0.6987	0.2678	0.9292	0.3597	0.2184	0.1650
0.20	−1.97	−1.98	−1.97	0.5690	0.3189	0.3365	0.3470	0.1703	0.1528
0.25	−2.00	−2.00	−1.98	0.3622	0.1194	0.1259	0.4500	0.0966	0.0914

The non‐adherence probability is set at 0.2. $\text{V}({\hat{\lambda }}_{\text{MLE}})$ denotes the sample variance of the maximum likelihood estimates, and $\text{V}({\hat{\lambda }}_{\text{2SLS}})$ denotes the sample variance of the two‐stage least square estimates.

LATE, local average treatment effect; RD, regression discontinuity.

Examining Table 1, where the non‐adherence probability is 0.1, we see that the two‐stage least squares and maximum likelihood methods for LATE estimation estimate the intervention effect (−2) in an unbiased manner for larger sample sizes (*N* = 500,1000and5000) and are mostly accurate and unbiased for the smallest sample size (*N* = 250). The Bayesian method is mostly unbiased for the largest sample size but is a little less accurate where *N* = 250,500or1000. This is an apparent drawback of the Bayesian approach and may be directly related to the vague prior beliefs used and a lack of data, particularly for small bandwidths and/or small sample sizes. In such scenarios, the use of stronger prior beliefs would be recommended, if appropriate.

In general, with the exception of the 0.05 bandwidth and a sample size of 250, the maximum likelihood‐based method for the variance estimation ( ${\hat{\nu }}_{\text{MLE}}$) has produced LATE variance estimates that lie close to the sample variance value of the calculated LATE estimates across the 200 samples, for each bandwidth and each sample size, suggesting that this method provides an accurate approximation for the LATE variance where the number of data points included in the analysis permits. The adjusted two‐stage least squares approach (9) ( ${\hat{\nu }}_{\text{2SLS}}^{\text{adj}}$) also estimates the LATE variance fairly accurately, with the exception of the 0.05 bandwidth and a sample size of 250, when comparing ${\hat{\nu }}_{\text{2SLS}}^{\text{adj}}$ and $V({\hat{\lambda }}_{\text{2SLS}})$. Conversely, the two‐stage least squares standard approach (6) ( ${\hat{\nu }}_{\text{2SLS}}^{\text{std}}$) consistently overestimates the LATE variance.

We see that the variance estimates using the Bayesian approach tend to be larger than those calculated using either the maximum likelihood method or the two‐stage least squares approach, except for some smaller bandwidths where the sample size is large, implying that the Bayesian approach to LATE estimation results in a less precise estimate of the LATE at the threshold. The inclusion of information from stronger prior knowledge/assumptions may help to alleviate this when using a Bayesian approach with real data.

Where *N* = 250 and the bandwidth is 0.05, all methods yielded large estimates of the LATE variance at the threshold. With such small datasets and a bandwidth of 0.05, implying that only a small fraction of the data are actually used in the corresponding RD design analysis, it is likely that *n*_*a*_+*n*_*b*_ will be particularly small and this may result in estimation and/or convergence problems. Notably, there were a small minority of simulated datasets for which the LATE estimate differed substantially from −2 and the corresponding estimated variance was large for both the two‐stage least squares and maximum likelihood approaches.

The results for the larger non‐adherence probability of *p* = 0.2(Table 2) generally show a similar pattern to those where the non‐adherence probability is 0.1. However, we note that the biases in the estimates of the LATE and the variance estimates are larger, for all methods, compared with those where the non‐adherence probability is 0.1. We would expect this because the RD design is less precise as the fuzziness of the data increases.

In general, ${\hat{\nu }}_{\text{MLE}}$ and $\text{V}({\hat{\lambda }}_{\text{MLE}})$ are similar, and ${\hat{\nu }}_{\text{2SLS}}^{\text{adj}}$ and $\text{V}({\hat{\lambda }}_{\text{2SLS}})$ are similar for most bandwidths and dataset sizes and for each of the chosen non‐adherence probabilities. This suggests that the likelihood‐based approach taken to LATE variance estimation accurately reflects the uncertainty in estimate of the LATE and that, when adopting a two‐stage least squares approach, it is advisable to use the adjusted method for variance estimation at the threshold. The Bayesian approach to variance estimation performed reasonably well for larger dataset sizes, but there was less certainty concerning the estimates for smaller datasets, particularly where the chosen bandwidth is also small. We now present an RD design analysis involving real data on the prescription of statins in UK primary care.

## 5 Example: prescription of statins in UK primary care

In the UK, the National Institute for Health and Care Excellence has issued guidelines that statins, a class of cholesterol‐lowering drugs, should be routinely prescribed, for the primary prevention of CVD, to adults aged under 75 whose 10‐year risk of experiencing a cardiovascular event (i.e. a stroke or myocardial infarction) exceeds 20% (NICE, 2008). Typically, 10‐year risk is calculated using an appropriate risk score function (for example, the Framingham risk score; Wilson et al., 1998). We consider a subset of patients from a large source of UK primary care data: The Health Improvement Network (THIN) (www.epic‐uk.org). The dataset consists of anonymized patient data collected at over 500 UK GP (General practitioner‐family doctor) practices. Of particular interest in this example are records pertaining to the prescription of statins to patients who are yet to experience a cardiovascular event.

We consider 10‐year risk score to be the assignment variable, and the intervention threshold is defined to be a 10‐year risk score greater than or equal to 20%. The ‘intervention’ is a statin prescription, and, because statins are prescribed to reduce LDL cholesterol, the outcome variable is the LDL cholesterol level in millimoles per litre (mmol/L). We use a subset of THIN data consisting of 1000 non‐diabetic male patients who were non‐smokers for whom risk scores were calculated between January 2007 and December 2008. Amongst these 1000 patients, there were 506 statin prescriptions during the period of observation.

**Figure 3:**
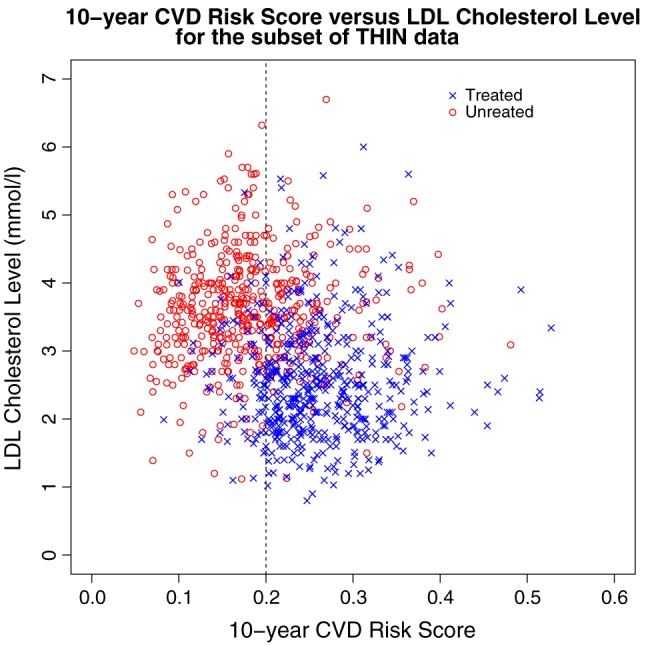
Scatter plot showing 10‐year cardiovascular disease (CVD) risk score versus low‐density lipoprotein (LDL) cholesterol level for The Health Improvement Network data subset. Patients who received statins are denoted ‘Treated’, and those who did not receive statins are denoted ‘Untreated’. The threshold (10‐year CVD risk score of 20%) is marked by a vertical dashed line.

Figure 3 shows a scatter plot of the 10‐year CVD risk score and the first recorded LDL cholesterol level measurement at least 1 month after risk score calculation for the 1000 men in the THIN data subset. Patients who received statins and those who did not are indicated by different coloured symbols. We see that there is some visual evidence of a discontinuity at the intervention threshold (a 10‐year CVD risk score of 20%), although there is obvious fuzziness in the data around this threshold. Nonetheless, this suggests that a fuzzy RD design could be applicable for these data, and we consider LATE estimation at the threshold as described in Section 4.

In this example, we define

$$\begin{array}{cc} Y_{i} & =\text{LDL cholesterol level (mmol/L)};\\ X_{i}^{c} & =(\text{10‐year CVD risk score})-0.2; \end{array}$$

and

$$\begin{array}{cc} Z_{i} & =\left\{\begin{array}{ll} 0 & \text{if}X_{i}^{c}<0;\\ 1 & \text{if}X_{i}^{c}\geq 0. \end{array}\right. \end{array}$$

$$\begin{array}{cc} T_{i} & =\left\{\begin{array}{ll} 0 & \text{if}i\text{th}\mathrm{patientdoesnotreceivestatins};\\ 1 & \text{if}i\text{th}\mathrm{patientdoesreceivestatins}. \end{array}\right. \end{array}$$

We consider the evaluation of the LATE at the statin intervention threshold ( $x_{i}^{c}=0.2$). As in Section 4, we considered five bandwidths (0.05, 0.1, 0.15, 0.2 and 0.25) for the RD design, and we used the same estimation methods for both the LATE and its variance, although with slightly different Bayesian priors (outlined in Section D of the Supporting Information). The empirical non‐adherence probabilities below and above the threshold were (0.26, 0.21, 0.18, 0.18, 0.18) and (0.35, 0.30, 0.28, 0.28, 0.28), respectively, for bandwidths (0.05, 0.10, 0.15, 0.20, 0.25). Table 3 shows the results of the RD analysis.

**Table 3:** Two‐stage least squares estimates ( ${\hat{\lambda }}_{\text{2SLS}}$), maximum likelihood estimates ( ${\hat{\lambda }}_{\text{MLE}}$) and Bayesian estimates ( ${\hat{\lambda }}_{\text{BAYES}}$) of the LATE for the effect of statins on LDL cholesterol level, together with corresponding variance estimates with a variety of chosen RD design bandwidths

Bandwidth (*h*)	${\hat{\lambda }}_{\text{2SLS}}$	${\hat{\lambda }}_{\text{MLE}}$	${\hat{\lambda }}_{\text{BAYES}}$	${\hat{\nu }}_{\text{2SLS}}^{\text{std}}$	${\hat{\nu }}_{\text{2SLS}}^{\text{adj}}$	${\hat{\nu }}_{\text{MLE}}$	${\hat{\nu }}_{\text{BAYES}}$
0.05	−3.66	−3.23	−1.70	1.4908	3.5536	3.8357	0.1921
0.10	−2.53	−2.50	−1.68	0.3384	0.4237	0.4150	0.1812
0.15	−2.11	−2.09	−1.75	0.1641	0.1733	0.1685	0.1829
0.20	−1.98	−1.99	−1.72	0.1187	0.1210	0.1216	0.1677
0.25	−2.00	−2.02	−1.74	0.1157	0.1187	0.1193	0.1312

The data used were taken from the THIN database.

LATE, local average treatment effect; RD, regression discontinuity; THIN, The Health Improvement Network.

Examining Table 3, we see that the two‐stage least squares and maximum likelihood methods yield similar intervention effect estimates for bandwidths 0.1 to 0.25. For bandwidth 0.05, the two‐stage least squares and maximum likelihood estimates differ more substantially, but we note that the corresponding variance estimates are also very large, which might be expected given the small bandwidth. For other bandwidths, ${\hat{\nu }}_{\text{2SLS}}^{\text{adj}}$ and ${\hat{\nu }}_{\text{MLE}}$ are similar, which may be expected given the results seen in the simulation study.

The Bayesian estimates of the LATE ( ${\hat{\lambda }}_{\text{BAYES}}$) are lower than both the likelihood‐based and two‐stage least squares estimates across all bandwidths. In addition, the Bayesian variance estimate is notably smaller than the other variance estimates for bandwidths 0.05 and 0.10 but larger than the other variance estimates for bandwidths 0.15 to 0.25. The choice of prior distributions may have contributed to the discrepancy between the Bayesian approach and the other non‐Bayesian approaches to LATE estimation at the threshold. Furthermore, we note that the empirical non‐compliance probabilities above and below the threshold in this example are higher than those used in the simulation studies of Section 4. Overall, though, all three methods indicated that the prescription of statins results in a reduction in LDL cholesterol level, which we would expect clinically.

## 6 Discussion

It has been shown that the LATE can be used to accurately estimate an intervention effect in an RD design at the intervention threshold. In essence, the LATE appears to be a relatively simple estimator, composed as a ratio of expectations from two linear models. In each of these models (for the numerator and for the denominator), a likelihood‐based approach may be taken for parameter estimation. This approach is fairly standard, but estimating the variance of the derived maximum likelihood estimator is less straightforward.

In this work, we have compared two‐stage least squares, likelihood‐based and Bayesian methods for estimating the LATE and its variance within a fuzzy RD design. We have shown that the maximum likelihood‐based method appears to accurately capture the variability in the LATE for a variety of RD design scenarios. Although a two‐stage least squares approach is often seen, at first sight, as an appropriate method for estimation in an RD design, we have shown that, whilst this approach yields a similar LATE estimate as that obtained using maximum likelihood methods, the standard variance estimate obtained using a two‐stage least squares approach is not always desirable and should be adjusted to ensure that the true variability of the LATE at the threshold is estimated. Using two‐stage least squares and adopting the standard approach to variance estimation resulted in an over‐estimation of the LATE variance.

The maximum likelihood‐based approach is best used where the outcome of interest is assumed to be normally distributed. For non‐normal outcomes, the maximum likelihood‐based approach may not be desirable, especially for small sample sizes or small bandwidths. In such scenarios, we would recommend either that a suitable transformation to a normally distributed outcome would be applicable, use of the central limit theorem be considered, or that the two‐stage least squares method be employed but with the adjusted method used to estimate the variance of the LATE at the threshold. The use of an approach where the variance estimation is overly conservative could be problematic if the results from an RD analysis were to be used to determine treatment allocation or perhaps for sample size calculations. It would be important to be mindful of this and perhaps consider the use of the maximum likelihood or the two‐stage least squares approach with the adjusted variance method in these scenarios.

An advantage of the other approaches over the Bayesian approach is that each requires less computation time and does not require prior beliefs to be specified, which may not always be appropriate in an RD design. In addition, with the maximum likelihood approach, we may exploit distributional assumptions in a more flexible manner using large‐sample properties of the maximum likelihood estimators derived. For smaller datasets and smaller bandwidths, the Bayesian approach can be problematic.

Another, alternative, method for calculating the LATE variance, with either the two‐stage least squares or maximum likelihood approaches, is bootstrapping. We note that this may sometimes be more computationally intensive than the two‐stage least squares and maximum likelihood methods presented in this paper, although not prohibitively so, but could provide a useful method for the checking of a chosen variance estimation method under a variety of possible modelling assumptions.

We note that this work has considered RD designs where the outcome of interest is continuous. At present, methodology concerning RD designs for non‐continuous outcomes (e.g. binary, count data and time‐to‐event data) is under‐developed, and the extension of this work to non‐continuous outcomes is an ongoing research area (for example, Bor et al., 2014).

To summarize, we have considered three approaches to estimation of the LATE within a typical RD design on a continuous outcome and associated methods for the estimation of the LATE variance, demonstrating the methods considered using both simulated and applied examples. We saw that two‐stage least squares approach is appropriate for unbiased estimation of the LATE but some properties of the standard variance estimator from the two‐stage least squares approach were less desirable in some cases. The likelihood‐based approach derived in Section 3 produced an unbiased estimator for the LATE and appeared to yield efficient estimates of the LATE variance that captured the true variability of the LATE estimator. The Bayesian approach tended to provide similar estimates of the LATE to the two‐stage least squares and likelihood‐based methods, although perhaps not for small design bandwidths, but represents an alternative approach in which available prior information can be incorporated into an RD design.

We are hopeful that RD designs will be used more widely in medicine, especially with the increasing use of electronic observational healthcare data. We recommend that, with an increase in the use of this methodology, due care is taken to ensure that the variability surrounding important estimators, such as the LATE, is estimated accurately.

## Supporting information

supporting Info Item
