# Supplementary material for: Approaches to the Estimation of the Local Average Treatment Effect in a Regression Discontinuity Design
Source: Scand Stat Theory Appl. 2016 Mar 22;43(4):978–95. doi: 10.1111/sjos.12224 (PMC5111792; doi:10.1111/sjos.12224)
Supplement: Supplementary file 1 — supporting Info Item [file SJOS-43-978-s001.pdf]

Supporting Information for 'Approaches to the Estimation of the Local Average Treatment Effect in a Regression Discontinuity Design'

Aidan G. O'Keeffe*[1] and Gianluca Baio[1]

[1]*Department of Statistical Science, University College London, Gower St., London, WC1E 6BT, UK.*

A Derivation of the LATE in an RD Design

Within an RD design with pre-specified bandwidth, h, we consider:

- An assignment variable: X;
- A binary threshold indicator: Z;
- A binary intervention indicator: T;
- A continuous outcome of interest: Y;
- A set of confounders (both observed and unobserved): $\mathcal{C}$.

For the RD design to be valid around the threshold, we make the assumption that the outcome Y is independent of Z, conditional on X, T and $\mathcal{C}$. Then at the threshold (i.e. for a specified

*E-mail address: a.o'keeffe@ucl.ac.uk; Corresponding author

value of X):

$$
\begin{aligned}
\mathbb{E}\left[Y|Z=1,\mathcal{C}\right]-\mathbb{E}\left[Y|Z=0,\mathcal{C}\right] = {} & \mathbb{E}\left[Y|Z=1,T=1,\mathcal{C}\right]\mathbb{P}\left(T=1|Z=1,\mathcal{C}\right) \\
& + \mathbb{E}\left[Y|Z=1,T=0,\mathcal{C}\right]\mathbb{P}\left(T=0|Z=1,\mathcal{C}\right) \\
& - \left\{\mathbb{E}\left[Y|Z=0,T=1,\mathcal{C}\right]\mathbb{P}\left(T=1|Z=0,\mathcal{C}\right)\right. \\
& \left. + \mathbb{E}\left[Y|Z=0,T=0,\mathcal{C}\right]\mathbb{P}\left(T=0|Z=0,\mathcal{C}\right)\right\}.
\end{aligned}
$$

Since Y is independent of Z, conditional on X, T and $\mathcal{C}$, we may write

$$
\begin{aligned}
\mathbb{E}\left[Y|Z=1,\mathcal{C}\right]-\mathbb{E}\left[Y|Z=0,\mathcal{C}\right] &= \mathbb{E}\left[Y|T=1,\mathcal{C}\right]\mathbb{P}\left(T=1|Z=1,\mathcal{C}\right)+\mathbb{E}\left[Y|T=0,\mathcal{C}\right]\mathbb{P}\left(T=0|Z=1,\mathcal{C}\right) \\
&\quad - \left\{\mathbb{E}\left[Y|T=1,\mathcal{C}\right]\mathbb{P}\left(T=1|Z=0,\mathcal{C}\right)+\mathbb{E}\left[Y|T=0,\mathcal{C}\right]\mathbb{P}\left(T=0|Z=0,\mathcal{C}\right)\right\} \\
&= \mathbb{E}\left[Y|T=1,\mathcal{C}\right]\mathbb{P}\left(T=1|Z=1,\mathcal{C}\right)+\mathbb{E}\left[Y|T=0,\mathcal{C}\right]\left\{1-\mathbb{P}\left(T=1|Z=1,\mathcal{C}\right)\right\} \\
&\quad - \left\{\mathbb{E}\left[Y|T=1,\mathcal{C}\right]\mathbb{P}\left(T=1|Z=0,\mathcal{C}\right)+\mathbb{E}\left[Y|T=0,\mathcal{C}\right]\left\{1-\mathbb{P}\left(T=1|Z=0,\mathcal{C}\right)\right\}\right\} \\
&= \left\{\mathbb{E}\left[Y|T=1,\mathcal{C}\right]-\mathbb{E}\left[Y|T=0,\mathcal{C}\right]\right\}\left\{\mathbb{P}\left(T=1|Z=1,\mathcal{C}\right)-\mathbb{P}\left(T=1|Z=0,\mathcal{C}\right)\right\}.
\end{aligned}
$$

Under the assumption that subjects are balanced with respect to confounders $\mathcal{C}$, above and below the threshold, we may write

$$
\mathbb{E}\left[Y|Z=1\right]-\mathbb{E}\left[Y|Z=0\right]=\left\{\mathbb{E}\left[Y|T=1\right]-\mathbb{E}\left[Y|T=0\right]\right\}\left\{\mathbb{P}\left(T=1|Z=1\right)-\mathbb{P}\left(T=1|Z=0\right)\right\}.
$$

Re-arranging

$$
\mathbb{E}\left[Y|T=1\right]-\mathbb{E}\left[Y|T=0\right]=\frac{\mathbb{E}\left[Y|Z=1\right]-\mathbb{E}\left[Y|Z=0\right]}{\mathbb{P}\left(T=1|Z=1\right)-\mathbb{P}\left(T=1|Z=0\right)}
$$

and the LATE, at the threshold, is recovered.

B Derivation of the Variance of the LATE

The maximum likelihood estimator for the LATE is given by

$$\hat{\lambda} = \frac{\hat{\beta}}{\hat{\pi}}. \tag{1}$$

We consider the variance for $\hat{\beta}$

$$\begin{aligned}\mathrm{Var}(\hat{\beta}) &= \mathrm{Var}(\hat{\beta}_{0a} - \hat{\beta}_{0b}) \\ &= \mathrm{Var}(\hat{\beta}_{0a}) + \mathrm{Var}(\hat{\beta}_{0b}).\end{aligned}$$

The estimators $\hat{\beta}_{0a}$ and $\hat{\beta}_{0b}$ are those of the intercept term in a normal linear model and these have the following form, conditional on $\mathbf{x} = (x_1, \ldots, x_n)^\top$, where the elements of $\mathbf{x}$ are centred about the threshold (x^c notation is removed for convenience).

$$\begin{aligned}\hat{\beta}_{0a} &= \bar{Y}_a - \hat{\beta}_{1a}\bar{x}_a \\ \hat{\beta}_{0b} &= \bar{Y}_b - \hat{\beta}_{1b}\bar{x}_b\end{aligned}$$

where

$$\begin{aligned}\bar{x}_a &= \frac{1}{n_a}\sum_{i\in\mathcal{A}} x_i \\ \bar{x}_b &= \frac{1}{n_b}\sum_{i\in\mathcal{B}} x_i \\ \bar{Y}_a &= \frac{1}{n_a}\sum_{i\in\mathcal{A}} Y_i \\ \bar{Y}_b &= \frac{1}{n_b}\sum_{i\in\mathcal{B}} Y_i.\end{aligned}$$

Furthermore, the maximum likelihood estimators for β_{1a} and β_{1b} are given by

$$\hat{\beta}_{1a} = \frac{\sum_{i\in\mathcal{A}}(x_{ia} - \bar{x}_a)(Y_{ia} - \bar{Y}_a)}{\sum_{i\in\mathcal{A}}(x_{ia} - \bar{x}_a)^2}$$
$$\hat{\beta}_{1b} = \frac{\sum_{i\in\mathcal{B}}(x_{ib} - \bar{x}_b)(Y_{ib} - \bar{Y}_b)}{\sum_{i\in\mathcal{B}}(x_{ib} - \bar{x}_b)^2}.$$

We make the following definitions

$$S_{xx}^{\mathcal{A}} = \sum_{i\in\mathcal{A}}(x_{ia} - \bar{x}_a)^2$$
$$S_{xx}^{\mathcal{B}} = \sum_{i\in\mathcal{B}}(x_{ib} - \bar{x}_b)^2$$

and, hence, we write $\hat{\beta}_{0a}$ and $\hat{\beta}_{0b}$ as

$$\begin{aligned}
\hat{\beta}_{0a} &= \bar{Y}_a - \frac{\bar{x}_a}{S_{xx}^{\mathcal{A}}}\left(\sum_{i\in\mathcal{A}} x_i Y_i - n_a \bar{x}_a \bar{Y}_a\right) \\
&= \frac{1}{n_a}\sum_{i\in\mathcal{A}} Y_i - \frac{\bar{x}_a}{S_{xx}^{\mathcal{A}}}\left(\sum_{i\in\mathcal{A}} x_i Y_i - \bar{x}_a \sum_{i\in\mathcal{A}} Y_i\right) \\
&= \sum_{i\in\mathcal{A}}\left(\frac{1}{n_a} + \frac{1}{S_{xx}^{\mathcal{A}}}(\bar{x}_a^2 - \bar{x}_a x_i)\right) Y_i \\
&= \sum_{i\in\mathcal{A}} a_i Y_i
\end{aligned}$$

with

$$a_i = \frac{1}{n_a} + \frac{1}{S_{xx}^{\mathcal{A}}}(\bar{x}_a^2 - \bar{x}_a x_i).$$

Similarly, for $i \in \mathcal{B}$ we may write

$$\hat{\beta}_{0b} = \sum_{i\in\mathcal{B}} b_i Y_i$$

where

$$b_i = \frac{1}{n_b} + \frac{1}{S_{xx}^{\mathcal{B}}}(\bar{x}_b^2 - \bar{x}_b x_i).$$

Hence, we may write

$$\hat{\beta} = \sum_{i\in\mathcal{A}} a_i Y_i - \sum_{i\in\mathcal{B}} b_i Y_i \qquad (2)$$

as the maximum likelihood estimator of the LATE numerator. If Y_i is normally distributed then the estimator for $\hat{\beta}$ in (2) is the sum of independent normal random variables and thus is normally distributed. If not, standard asymptotic results apply and $\hat{\beta}$ is approximately normally distributed for large samples.

Assuming that $\text{Var}(Y_i) = \sigma^2$ is constant for all $i \in \mathcal{A} \cup \mathcal{B}$ and that the outcomes Y are independent for each individual. Then

$$\begin{aligned}\text{Var}(\hat{\beta}) &= \text{Var}\left(\sum_{i\in\mathcal{A}} a_i Y_i - \sum_{i\in\mathcal{B}} b_i Y_i\right)\\ &= \sum_{i\in\mathcal{A}} a_i^2 \text{Var}(Y_i) + \sum_{i\in\mathcal{B}} b_i^2 \text{Var}(Y_i)\\ &= \sigma^2 \left(\sum_{i\in\mathcal{A}} a_i^2 + \sum_{i\in\mathcal{B}} b_i^2\right).\end{aligned}$$

In this work, we will assume that $\text{Var}(Y_i)$ is the same for subjects above and below the threshold. However, we could relax this assumption and, for example, set $\text{Var}(Y_i) = \sigma_a^2$ above the threshold and $\text{Var}(Y_i) = \sigma_b^2$ below the threshold. In this case

$$\text{Var}(\hat{\beta}) = \sigma_a^2 \sum_{i\in\mathcal{A}} a_i^2 + \sigma_b^2 \sum_{i\in\mathcal{B}} b_i^2.$$

We form similar linear models for T_i, above and below the threshold, of the form

$$\begin{aligned}T_i &= \pi_{0a} + \pi_{1a} x_i + \omega_{ai} \quad \text{for } i \in \mathcal{A}\\ T_i &= \pi_{0b} + \pi_{1b} x_i + \omega_{bi} \quad \text{for } i \in \mathcal{B}\end{aligned}$$

with ω_{ai} and ω_{bi} normal mean-zero error terms such that $\text{Var}(\omega_{ai}) = \phi_a^2$ and $\text{Var}(\omega_{bi}) = \phi_b^2$. In a similar manner to the linear models for Y_i, the maximum likelihood estimates for π_{0a} and π_{0b} are written

$$\begin{aligned}\hat{\pi}_{0a} &= \sum_{i\in\mathcal{A}} a_i T_i;\\ \hat{\pi}_{0b} &= \sum_{i\in\mathcal{B}} b_i T_i.\end{aligned}$$

Then $\hat{\pi}$ is written

$$\begin{aligned}\hat{\pi} &= \hat{\pi}_{0a} - \hat{\pi}_{0b} \\ &= \sum_{i\in\mathcal{A}} a_i T_i - \sum_{i\in\mathcal{B}} b_i T_i\end{aligned}$$

and, for large samples, $\hat{\pi}$ is approximately normally distributed with

$$\mathrm{Var}(\hat{\pi}) = \phi_a^2 \sum_{i\in\mathcal{A}} a_i^2 + \phi_b^2 \sum_{i\in\mathcal{B}} b_i^2.$$

Hence, we argue that $(\hat{\beta}, \hat{\pi})^\top$ will have, approximately, a bivariate normal distribution. We consider $\mathrm{Cov}(\hat{\beta}, \hat{\pi})$.

$$\begin{aligned}\mathrm{Cov}(\hat{\beta}, \hat{\pi}) &= \mathrm{Cov}\left(\sum_{i\in\mathcal{A}} a_i Y_i - \sum_{i\in\mathcal{B}} b_i Y_i, \sum_{i\in\mathcal{A}} a_i T_i - \sum_{i\in\mathcal{B}} b_i T_i\right) \\ &= \mathbb{E}\left[\left(\sum_{i\in\mathcal{A}} a_i Y_i - \sum_{i\in\mathcal{B}} b_i Y_i\right)\left(\sum_{i\in\mathcal{A}} a_i T_i - \sum_{i\in\mathcal{B}} b_i T_i\right)\right] - (\beta_{0a} - \beta_{0b})(\pi_{0a} - \pi_{0b}) \\ &= \mathbb{E}\left[\left(\sum_{i\in\mathcal{A}} a_i Y_i\right)\left(\sum_{i\in\mathcal{A}} a_i T_i\right)\right] + \mathbb{E}\left[\left(\sum_{i\in\mathcal{B}} b_i Y_i\right)\left(\sum_{i\in\mathcal{B}} b_i T_i\right)\right] \\ &\quad - \mathbb{E}\left[\left(\sum_{i\in\mathcal{A}} a_i Y_i\right)\left(\sum_{i\in\mathcal{B}} b_i T_i\right)\right] - \mathbb{E}\left[\left(\sum_{i\in\mathcal{B}} b_i Y_i\right)\left(\sum_{i\in\mathcal{A}} a_i T_i\right)\right] \\ &\quad + \pi_{0a}\beta_{0b} + \pi_{0b}\beta_{0a} - \pi_{0a}\beta_{0a} - \pi_{0b}\beta_{0b} \\ &= \mathbb{E}\left[\left(\sum_{i\in\mathcal{A}} a_i Y_i\right)\left(\sum_{i\in\mathcal{A}} a_i T_i\right)\right] + \mathbb{E}\left[\left(\sum_{i\in\mathcal{B}} b_i Y_i\right)\left(\sum_{i\in\mathcal{B}} b_i T_i\right)\right] \\ &\quad - \pi_{0a}\beta_{0a} - \pi_{0b}\beta_{0b}\end{aligned}$$

We note that

$$\mathbb{E}\left[\left(\sum_{i\in\mathcal{A}} a_i Y_i\right)\left(\sum_{i\in\mathcal{A}} a_i T_i\right)\right] = \sum_{j=1}^{n_a}\sum_{i=1}^{n_a} \mathbb{1}(i \neq j) a_i a_j \mathbb{E}(Y_i)\mathbb{E}(T_j) + \sum_{i=1}^{n_a} a_i^2 \mathbb{E}(Y_i T_i) \tag{3}$$

Assume that the covariance between Y_i and T_i may be written

$$\mathrm{Cov}(Y_i, T_i) = \begin{cases} \rho_a & \text{if } i \in \mathcal{A} \\ \rho_b & \text{if } i \in \mathcal{B} \end{cases}$$

then, for $i \in \mathcal{A}$

$$\begin{aligned} \mathrm{Cov}(Y_i, T_i) &= \mathbb{E}(Y_i T_i) - \mathbb{E}(Y_i)\mathbb{E}(T_i) \\ \implies \rho_a &= \mathbb{E}(Y_i T_i) - \mathbb{E}(Y_i)\mathbb{E}(T_i). \end{aligned}$$

Rearranging, we see that

$$\mathbb{E}(Y_i T_i) = \rho_a + \mathbb{E}(Y_i)\mathbb{E}(T_i).$$

Hence, by substitution into Equation 3, we obtain

$$\begin{aligned} \mathbb{E}\left[\left(\sum_{i\in\mathcal{A}} a_i Y_i\right)\left(\sum_{i\in\mathcal{A}} a_i T_i\right)\right] &= \rho_a \sum_{i\in\mathcal{A}} a_i^2 + \sum_{i\in\mathcal{A}}\sum_{j\in\mathcal{A}} a_i a_j \mathbb{E}(Y_i)\mathbb{E}(T_j) \\ &= \rho_a \sum_{i\in\mathcal{A}} a_i^2 + \sum_{i\in\mathcal{A}}\sum_{j\in\mathcal{A}} \mathbb{E}(a_i Y_i)\mathbb{E}(a_j T_j) \\ &= \rho_a \sum_{i\in\mathcal{A}} a_i^2 + \pi_{0a}\beta_{0a} \end{aligned}$$

Similarly,

$$\mathbb{E}\left[\left(\sum_{i\in\mathcal{B}} b_i Y_i\right)\left(\sum_{i\in\mathcal{B}} b_i T_i\right)\right] = \rho_b \sum_{i=1}^{n_b} b_i^2 + \pi_{0b}\beta_{0b}.$$

Therefore

$$\mathrm{Cov}(\hat{\beta}, \hat{\pi}) = \rho_a \sum_{i=1}^{n_a} a_i^2 + \rho_b \sum_{i=1}^{n_b} b_i^2.$$

Then, the distribution of $(\hat{\beta}, \hat{\pi})^\top$ is

$$\begin{pmatrix} \hat{\beta} \\ \hat{\pi} \end{pmatrix} \sim \mathcal{N}\left(\begin{pmatrix} \beta \\ \pi \end{pmatrix}, \begin{pmatrix} \sigma^2\left(\sum_{i\in\mathcal{A}} a_i^2 + \sum_{i\in\mathcal{B}} b_i^2\right) & \rho_a \sum_{i\in\mathcal{A}} \frac{a_i}{n_a} + \rho_b \sum_{i\in\mathcal{B}} \frac{b_i}{n_b} \\ \rho_a \sum_{i\in\mathcal{A}} a_i^2 + \rho_b \sum_{i\in\mathcal{B}} b_i^2 & \phi_a^2 \sum_{i\in\mathcal{A}} a_i^2 + \phi_b^2 \sum_{i\in\mathcal{B}} b_i^2 \end{pmatrix}\right).$$

We write this as:

$$\begin{pmatrix} \hat{\beta} \\ \hat{\pi} \end{pmatrix} \sim \mathcal{N}\left(\begin{pmatrix} \beta \\ \pi \end{pmatrix}, \begin{pmatrix} \Gamma_{11} & \Gamma_{12} \\ \Gamma_{12} & \Gamma_{22} \end{pmatrix}\right).$$

Our interest lies in determining $\text{Var}(\hat{\beta}/\hat{\pi})$. We have derived an approximate joint density for $(\hat{\beta}, \hat{\pi})^\top$. We set

$$\lambda = g(\beta, \pi) = \frac{\beta}{\pi}$$

and consider a Taylor expansion (i.e. a multivariate delta method) to derive an approximation for $\text{Var}(\hat{\lambda})$. The delta method approximation for the variance of $\hat{\lambda}$ is given by:

$$\begin{aligned}
\text{Var}(\hat{\lambda}) &= [\nabla g(\hat{\beta}, \hat{\pi})]\text{Cov}(\hat{\beta}, \hat{\pi})[\nabla g(\hat{\beta}, \hat{\pi})]^\top \\
&= \begin{pmatrix} \frac{1}{\hat{\pi}} & -\frac{\hat{\beta}}{\hat{\pi}^2} \end{pmatrix} \begin{pmatrix} \Gamma_{11} & \Gamma_{12} \\ \Gamma_{12} & \Gamma_{22} \end{pmatrix} \begin{pmatrix} \frac{1}{\hat{\pi}} \\ -\frac{\hat{\beta}}{\hat{\pi}^2} \end{pmatrix} \\
&= \frac{1}{\hat{\pi}^2}\Gamma_{11} + \frac{\hat{\beta}^2}{\hat{\pi}^4}\Gamma_{22} - \frac{2\hat{\beta}}{\hat{\pi}^3}\Gamma_{12} \\
&= \frac{\sigma^2}{\hat{\pi}^2}\left(\sum_{i\in\mathcal{A}} a_i^2 + \sum_{i\in\mathcal{B}} b_i^2\right) + \frac{\hat{\beta}^2}{\hat{\pi}^4}\left(\phi_a^2 \sum_{i\in\mathcal{A}} a_i^2 + \phi_b^2 \sum_{i\in\mathcal{B}} b_i^2\right) \\
&\quad - \frac{2\hat{\beta}}{\hat{\pi}^3}\left(\rho_a \sum_{i\in\mathcal{A}} a_i^2 + \rho_b \sum_{i\in\mathcal{B}} b_i^2\right).
\end{aligned}$$

Since σ^2, ρ_a, ρ_b are unknown, we estimate these parameters as follows

$$\begin{aligned}
\hat{\rho}_a &= \frac{1}{n_a - 1}\sum_{i\in\mathcal{A}} (y_i - \hat{y}_i)\left(t_i - \hat{t}_i\right) \\
\hat{\rho}_b &= \frac{1}{n_b - 1}\sum_{i\in\mathcal{B}} (y_i - \hat{y}_i)\left(t_i - \hat{t}_i\right) \\
\hat{\phi}_a^2 &= \frac{1}{n_a - 2}\sum_{i\in\mathcal{A}} \left(t_i - \hat{t}_i\right)^2 \\
\hat{\phi}_b^2 &= \frac{1}{n_b - 2}\sum_{i\in\mathcal{B}} \left(t_i - \hat{t}_i\right)^2 \\
\hat{\sigma}^2 &= \frac{(n_a - 2)s_a^2 + (n_b - 2)s_b^2}{n_a + n_b - 4}.
\end{aligned}$$

Here, s_a^2 and s_b^2 denote the sample variance values

$$s_a^2 = \frac{1}{n_a - 2} \sum_{i \in \mathcal{A}} (y_i - \hat{y}_i)^2$$
$$s_b^2 = \frac{1}{n_b - 2} \sum_{i \in \mathcal{B}} (y_i - \hat{y}_i)^2$$

from the linear models ((10) in Section 3.2 of the paper). The terms $\hat{y}_i$ and $\hat{t}_i$ denote fitted values from the corresponding models for the ith individual. Note that $\hat{\rho}_a$ and $\hat{\rho}_b$ denote the sample covariance between Y_i and T_i for the populations above and below the threshold, respectively. Thus, our estimate for the variance of the LATE is

$$\begin{aligned}\text{Var}(\hat{\lambda}) = &\frac{\hat{\sigma}^2}{\hat{\pi}^2} \left(\sum_{i \in \mathcal{A}} a_i^2 + \sum_{i \in \mathcal{B}} b_i^2 \right) + \frac{\hat{\beta}^2}{\hat{\pi}^4} \left(\hat{\phi}_a^2 \sum_{i \in \mathcal{A}} a_i^2 + \hat{\phi}_b^2 \sum_{i \in \mathcal{B}} b_i^2 \right) \\ &- \frac{2\hat{\beta}^2}{\hat{\pi}^3} \left(\hat{\rho}_a \sum_{i \in \mathcal{A}} a_i^2 + \hat{\rho}_b \sum_{i \in \mathcal{B}} b_i^2 \right).\end{aligned}$$

C Bayesian Priors for the Simulation Study

We assume the following prior distributions for the parameters of interest:

$$\begin{aligned} &\beta_{0a} \sim \mathcal{N}(3, 4) && \beta_{0b} \sim \mathcal{N}(5, 4) \\ &\beta_{1a} \sim \mathcal{N}(0.3, 4) && \beta_{1b} \sim \mathcal{N}(0.5, 4) \\ &\gamma_0 \sim \mathcal{N}(2.1, 1) && \gamma_1 \sim \mathcal{N}(0, 10) \\ &\gamma_2 \sim \mathcal{N}(-2.1, 1) && \gamma_3 \sim \mathcal{N}(0, 10) \\ &\sigma \sim \mathcal{U}(0, 5). \end{aligned}$$

D Bayesian Priors for the Analysis of THIN Data

We assume the following prior distributions for the parameters of interest:

$$
\begin{aligned}
&\beta_{0a} \sim \mathcal{N}(2,4) && \beta_{0b} \sim \mathcal{N}(4,4) \\
&\beta_{1a} \sim \mathcal{N}(0,4) && \beta_{1b} \sim \mathcal{N}(0,4) \\
&\gamma_0 \sim \mathcal{N}(1,10) && \gamma_1 \sim \mathcal{N}(0,20) \\
&\gamma_2 \sim \mathcal{N}(-1,10) && \gamma_3 \sim \mathcal{N}(0,20) \\
&\sigma \sim \mathcal{U}(0,5).
\end{aligned}
$$
